# Opening and changing: mammalian SWI/SNF complexes in organ development and carcinogenesis

**DOI:** 10.1098/rsob.240039

**Published:** 2024-10-30

**Authors:** Fadia Abu Sailik, Bright Starling Emerald, Suraiya Anjum Ansari

**Affiliations:** ^1^ Department of Biochemistry and Molecular Biology, College of Medicine and Health Sciences, United Arab Emirates University, Al Ain, Abu Dhabi, UAE; ^2^ Department of Anatomy, College of Medicine and Health Sciences, United Arab Emirates University, Al Ain, Abu Dhabi, UAE; ^3^ Zayed Center for Health Sciences, United Arab Emirates University, Al Ain, Abu Dhabi, UAE; ^4^ ASPIRE Precision Medicine Research Institute Abu Dhabi (PMRI-AD), United Arab Emirates University, Al Ain, Abu Dhabi, UAE

**Keywords:** chromatin remodelling, cancer, gene expression, cell fate

## Abstract

The switch/sucrose non-fermentable (SWI/SNF) subfamily are evolutionarily conserved, ATP-dependent chromatin-remodelling complexes that alter nucleosome position and regulate a spectrum of nuclear processes, including gene expression, DNA replication, DNA damage repair, genome stability and tumour suppression. These complexes, through their ATP-dependent chromatin remodelling, contribute to the dynamic regulation of genetic information and the maintenance of cellular processes essential for normal cellular function and overall genomic integrity. Mutations in SWI/SNF subunits are detected in 25% of human malignancies, indicating that efficient functioning of this complex is required to prevent tumourigenesis in diverse tissues. During development, SWI/SNF subunits help establish and maintain gene expression patterns essential for proper cellular identity and function, including maintenance of lineage-specific enhancers. Moreover, specific molecular signatures associated with SWI/SNF mutations, including disruption of SWI/SNF activity at enhancers, evasion of G0 cell cycle arrest, induction of cellular plasticity through pro-oncogene activation and Polycomb group (PcG) complex antagonism, are linked to the initiation and progression of carcinogenesis. Here, we review the molecular insights into the aetiology of human malignancies driven by disruption of the SWI/SNF complex and correlate these mechanisms to their developmental functions. Finally, we discuss the therapeutic potential of targeting SWI/SNF subunits in cancer.

## 1. Introduction

Packaging eukaryotic DNA into chromatin allows the genome to be categorized into transcriptionally active and repressed regions. Different partitioning patterns enable distinct transcriptional programmes to emerge from the same gene sequence. The formation of distinct chromatin states during development and their propagation during the distinct actions of transcription, DNA replication and DNA repair requires dynamic chromatin structural rearrangements [1]. Chromatin is a multidimensional and hierarchical nucleoprotein complex that includes both histones and non-histone proteins. The nucleosome, made up of nucleosome core and linker region, is the main structural component of chromatin, which is an octameric structure composed of two molecules, each of the core histones H2A, H2B, H3 and H4, and contains 147 base pairs of DNA wrapped around the histone octamer [2].

In the early 1960s, Vincent Allfrey pioneered the ground-breaking discovery that histones undergo post-translational changes (PTMs) [3]. This, followed by the determination of the high-resolution X-ray structure of the nucleosome in 1997 [2], provided information on how these alterations could affect the chromatin structure. After seeing how the highly basic histone amino (N)-terminal tails extend from their nucleosomes and interact with nearby nucleosomes, it became clear that modifying these tails would affect inter-nucleosomal interactions and, in turn, modulate chromatin structure [4]. Furthermore, PTMs can also act as docking sites for proteins with particular structural domains; for instance, bromodomains recognize acetylated lysines, and chromodomains recognize methylated lysines, and the attraction or repulsion of these proteins affects subsequent processes [5]. The histone code hypothesis postulates that PTMs generate a code that may be read by the effector proteins [6].

Similarly, such changes in chromatin structure can also be produced by ATP-dependent chromatin remodellers, an evolutionarily conserved complexes that modulate nucleosome position to regulate a variety of DNA-templated mechanisms, including replication, repair and transcription [7,8]. Remodellers are multi-subunit complexes which can be divided into four subfamilies: Imitation switch (ISWI), chromodomain helicase DNA-binding (CHD), Inositol requiring 80 (INO80) and switch/sucrose non-fermentable (SWI/SNF), all of which have a catalytic ATPase domain alongside one or more accessory domains essential for their functional interactions with other proteins [9,10]. Remodellers collaborate with histone modification enzymes and site-specific transcription factors to reposition or expel histones and enable transcription factor binding to DNA [11]. Thus, chromatin remodellers play pivotal roles as essential regulators in nearly all chromosomal functions, and any disruption in their normal functioning can lead to various diseases, including developmental disorders and cancer [12,13].

Due to their key roles in regulating chromatin architecture and gene expression, SWI/SNF complexes are among the most widely investigated ATP-dependent chromatin remodelling complexes in development, differentiation and disease. Studies have identified mutations in SWI/SNF subunits in several human malignancies in the last decade. In 2013, two seminal studies extensively investigated cancer genome/exome sequencing data and found that around 20% of tumours have SWI/SNF subunit gene mutations [14,15]. For example, AT-rich interaction domain 1A (ARID1A) is a SWI/SNF subunit whose genetic mutations lead to functional impairment. It is the most frequently mutated subunit of the SWI/SNF complex, with inactivating mutations found in 6% of cancers, including ovarian clear cell cancers (45% of cases), uterine endometrioid cancers (37%) and hepatocellular carcinoma HCC (14%), highlighting the roles of ARID1A in cancer [16]. Similarly, AT-rich interaction domain 2 (ARID2) is an integral part of the chromatin remodelling complex, playing a critical role in many biological processes, including transcription regulation, cell cycle control, embryonic development and DNA damage repair. Loss-of-function mutations in the ARID2 protein are also common in many types of cancer, including HCC [17].

In this review, we thoroughly examine the SWI/SNF ATP-dependent chromatin-remodelling complexes, emphasizing its involvement in both organ development and carcinogenesis. We specifically underscore shared mechanisms between developmental disorders and carcinogenesis arising from mutations in SWI/SNF complex subunits and aim to identify potential treatment strategies by targeting chromatin remodelling complexes.

## 2. Switch/sucrose non-fermentable remodellers

The highly conserved SWI/SNF remodeller was first discovered in yeast when genes affecting the switch in mating type and sucrose non-fermenting (SNF) phenotypes were identified [18,19]. These remodellers influence histone–DNA interactions and play crucial roles in controlling chromatin structure and gene expression [20]. By sliding and expelling histone octamers, it hydrolyses ATP to change the structure of the chromatin, making DNA regions accessible to different transcriptional regulators, including repressors and activators [1]. The SWI/SNF complex is thought to regulate around 6% of the genes in yeast [21]. Recent studies of *Saccharomyces cerevisiae* SWI/SNF complex bound to a nucleosome using cryo-electron microscopy suggest that SNF2 helicase-SANT associated (HSA) domain bridges all modules in the structure, sandwiching the actin-related protein (Arp) module between the ATPase and the remaining components of the complex. SNF2, SNF5 and an asymmetric dimer of SWI3 make up the assembly scaffold found in the body. In the centre of SWI/SNF is a conserved subunit called Swi1, which serves as a molecular nexus [22].

Unlike yeast, the mammalian SWI/SNF complexes (canonical BAF (cBAF), polybromo-associated BAF complexes (PBAF)) appear in multiple forms with various subunit compositions. The ATPases that hydrolyse the ATP, BRG1 (SMARCA4) or BRM (SMARCA2) subunits are often included in these complexes along with 6–11 other proteins known as BRG1/BRM associated factors (BAFs), which are essential for binding to DNA or with other proteins [23]. In humans, three of these subunits, SMARCB1, SMARCC1 and SMARCC2, referred to as ‘core subunits’ together with SMARCA2 or SMARCA4, are needed for the ATP-dependent chromatin remodelling activity of the SWI/SNF complexes [24]. These complexes have been further divided into two categories based on their subunit composition: BAF complexes have either ARID1A or ARID1B subunits, whereas PBAF complexes have PBRM1 and ARID2 subunits, along with GLTSCR1-containing (GBAF) complex, which was identified recently and is also referred to as non-canonical BAF (ncBAF) complex [25].

**Figure 1.**
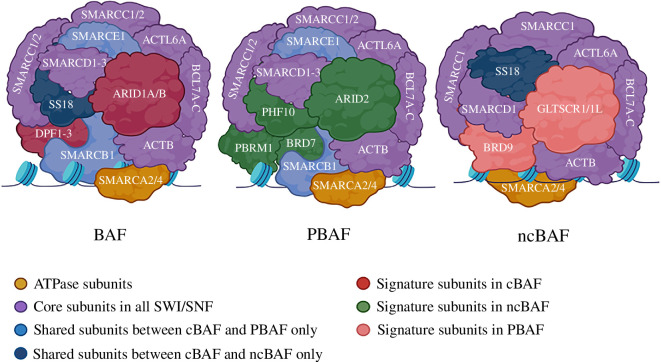
The mammalian SWI/SNF chromatin remodeller BAF, PBAF and ncBAF complexes. These involve two replaceable ATPase subunits (SMARCA2/4), core subunits (SMARCC1, SMARCD1, ACTL6A, BCL7A-C and ACTB) and a variety of signature subunits.

Moreover, cBAF and PBAF can each incorporate up to 15 subunits expressed by more than 29 genes, leading to more than 1400 potential combinations. These involve two replaceable core ATPase subunits, namely SMARCA4 (BRG1) or SMARCA2 (BRM), eight common auxiliary subunits, namely ACTB, ACTL6A, BCL7A, SMARCB1 (BAF47), SMARCD1 (BAF60A), SMARCE1 (BAF57) and two SMARCC1/2 (BAF155 and BAF170) [26], and a variety of lineage-restricted subunits, including canonical BAF-signature subunits ARID1A/B (BAF250A/B), DPF1/2/3 (BAF45B/D/C) and SS18, PBAF-signature subunits ARID2 (BAF200), PHF10 (BAF45A), PBRM1 (BAF180) and BRD7, and ncBAF-signature subunits GLTSCR1/GLTSCR1L and BRD9 (figure 1) [14]. This heterogeneity of the BAF/PBAF complexes in higher eukaryotes is thought to provide the functional diversity needed by the differentiated cells’ dynamic cellular requirements and specialized functions [27]. As revealed by the cryo-EM structure in recent studies in human cells, the nucleosome is sandwiched by the Base and ATPase modules. When ATP is hydrolysed, the ATPase motor, located close to the nucleosomal DNA, interacts with and facilitates DNA movement within the nucleosome [28].

## 3. Chromatin remodelling by SWI/SNF complexes: mechanism of action

The ability of SWI/SNF remodellers to cause histone octamers to ‘slide’ on the same DNA has been demonstrated. Histone octamers might be released from the DNA bound by SWI/SNF, thus allowing them to interact with other DNA molecules in trans. Rearranging the nucleosome positions within a promoter may lead to either activation or repression of gene expression since various promoters have distinct nucleosomal structures [29].

Early studies revealed that SWI/SNF remodelling induces a sequential disassembly of nucleosomes. Initially, an H2A/H2B dimer is rapidly displaced, succeeded by the delayed removal of an entire histone octamer. Notably, SWI/SNF-mediated nucleosome disassembly did not necessitate additional components such as chaperones or histone acceptors. Both single-molecule observations and mass measurements suggest a crucial step in this process involves the translocation of a nucleosome towards its adjacent counterpart. When recruited by the transcriptional activator Gal4-VP16, the SWI/SNF complex preferentially mobilizes the proximal nucleosome and destabilizes the neighbouring nucleosome [30].

**Figure 2.**
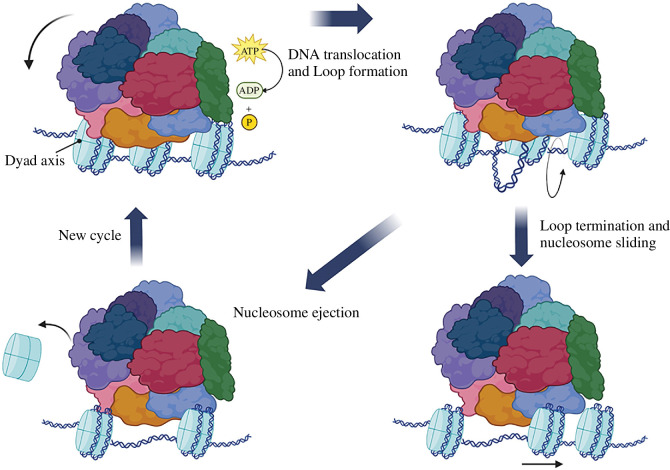
The mechanism of nucleosome remodelling dependent on SWI/SNF. After initially attaching to the nucleosome, the SWI/SNF complex uses the energy from ATP hydrolysis to shift DNA around the nucleosome. Nucleosomal DNA is engaged by the ATPase subunit around two turns away from the dyad. Using the energy from ATP hydrolysis, a transient DNA loop is created, disrupting the interaction between histones and DNA. This loop moves the DNA relative to the nucleosome as it slides around it. Consequently, this sliding can expel neighbouring nucleosomes, completing a remodelling cycle.

A simple model for remodelling would thus be where the SWI/ SNF complex utilizes the energy of ATP hydrolysis to slide DNA around the nucleosome after binding of the remodeller to the nucleosome in the initial step (figure 2). This binding occurs with nanomolar affinity [31]. The ATPase subunit engages nucleosomal DNA at a position about two turns away from the dyad. It disrupts histone–DNA interaction by generating a temporary DNA loop using the energy of ATP hydrolysis. Subsequently, the DNA loop propagates (slides) around the nucleosome, moving the DNA with respect to the nucleosome. Thus, adjacent nucleosomes may be ejected due to sliding following a remodelling cycle [1,32].

Recent studies have hypothesized that interactions between transcription factors and certain nucleosome remodellers are critical for transcription factor binding to their corresponding motifs. SWI/SNF remodellers facilitate transcription-factor binding by selective recruitment or by satisfying particular chromatin requirements rather than establishing global nucleosome dynamics, leading to factor-independent binding opportunities [33]. For example, it was found that BRG1 is required to bind pluripotency transcription factor Oct4, supporting the idea that SWI/SNF serves a role in transcription factor’s binding along with its capacity to eject nucleosomes *in vitro* [34]. Another study highlights the critical role of the actin-related BAP55 subunit in conferring enhancer accessibility and enabling the transcriptional response to the Notch signalling pathway in *Drosophila*, which is essential for many cell fate decisions during development by controlling various gene expression programmes via the transcription factor CSL, confirming that these remodelling mechanisms have a specific influence on transcription factor binding and downstream gene expression [33,35].

During mammalian embryogenesis, the auxiliary component of SWI/SNF-like BAF known as actin-like 6A (ACTL6A), also referred to as BAF53A, is essential for maintaining stem or progenitor cells [36,37]. It was also reported that ACTL6A regulates the repair of cisplatin-induced DNA damage in cancer cells via SWI/SNF-mediated remodelling, which is necessary for effective DNA repair [38]. Since nucleosomes prevent DNA repair, as shown in *in vitro* studies, SWI/SNF may affect other repair proteins, including Fanconi anaemia (FA), homologous recombination (HR) structure-specific endonucleases and gap-filling translesion DNA synthesis (TLS), to aid in the healing of cisplatin-DNA lesions [39].

The SWI/SNF complex is also necessary for the yeast replication origin, known as autonomous replication sequences (ARSs). These ARS regions encompass a replication enhancer sequence element, which is specifically bound by the transcription factor ABF1 [40]. The ARS121 mitotic mini-chromosome stability assay illustrates that the deactivation of SWI/SNF notably impairs the effective functioning of ARS121. This dependency on SWI/SNF is specific to the minimal ARS121 origin, distinguishing it from the broader ARS121, as it operates independently of the replication enhancer factor ABF1 [41]. Another study has proposed the incorporation of PBAF in repriming replication downstream of the replication fork halted by DNA damage. It was reported that the loss of the PBRM1 subunit led to a reduction in ubiquitination of proliferating cell nuclear antigen (PCNA) and the E3 ligase (Rad18) following UV exposure. This was associated with a slight reduction in fork progression, suggesting the role of PBAF in repriming replication downstream of the replication fork halted at DNA damage sites [42].

In summary, SWI/SNF chromatin remodellers are linked to a variety of cellular functions, including gene expression, DNA replication, DNA damage repair, genome stability and tumour suppression, as detailed in the following sections.

## 4. Recruitment of SWI/ SNF complex

The remodelling process can be divided into three stages. First, remodellers must be recruited to specific genomic regions in a sequence-specific manner at precise times. Once a target nucleosome is identified, remodellers engage in their remodelling activity, which may include nucleosome sliding and requires a specific movement orientation. Finally, after the nucleosome structure is disrupted, remodellers recognize the completion of the process and cease further activity [43].

In yeast and mammalian cells, interactions between Pol II holoenzyme and SWI/SNF suggest that remodellers might be recruited by sequence-specific activators via an activation domain rather than by promoter sequences, TBP or RNA Pol II [44].

**Figure 3.**
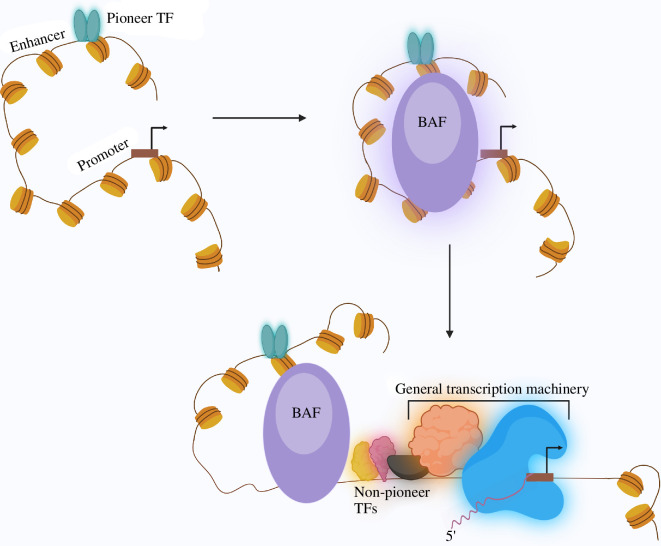
TF–BAF interaction to regulate transcription. One model of BAF-mediated transcriptional regulation involves BAF subunits interacting with pioneer transcription factors at enhancer sequences. This interaction leads to BAF engaging with nucleosomes at promoters, causing chromatin remodelling, recruiting non-pioneer transcription factors and assembling the general transcription complex at the promoter region.

Enhancers serve as platforms for transcription factor (TF) binding, targeting the transcriptional machinery to specified promoters to accelerate gene transcription [45]. The efficiency of transcription control in a cell-type-specific manner is conferred by the type, frequency and positioning of TF binding motifs within an enhancer [46]. Chromatin prevents many TFs from binding to regulatory sites across the genome, playing a regulatory role in selective gene expression [47]. Thus, enhancer function requires histone octamer displacement to generate nucleosome-free DNA suitable for TF engagement and activation of relevant epigenetic regulatory mechanisms. Overcoming the nucleosome barrier is essential for activating enhancers and restricting their activity to specific cell types [45]. For example, AP-1 TFs bind to nucleosome-blocked enhancers and recruit the BAF complex to drive nucleosome displacement, creating a permissive chromatin structure [48] (figure 3).

The transcription factors NANOG, SOX2 and OCT4 represent a well-established TF-cofactor inter-communication paradigm in mammals that leads to nucleosome disruption or rearrangement [49]. As a master regulator and pioneer factor, OCT4 binds to nucleosomal DNA, facilitating chromatin opening [50]. OCT4 also interacts with epigenetic regulatory components, particularly the SWI/SNF subunits SMARCC1, SMARCA4 and ARID1A [51]. Chromatin accessibility at OCT4-bound sites also depends on BRG1, which OCT4 recruits to facilitate non-pioneer transcription factor binding and the expression of pluripotency-related genes [52]. Recent research in mESCs highlights the role of RNA Polymerase II (RNAPII), BAF and sequence-specific TFs, showing that RNAPII promoter-proximal pausing stabilizes BAF occupancy and enhances nucleosome eviction, while pluripotency transcription factor binding ensures locus-specific remodelling by BAF [53]. Cooperation between BAF and histone acetyltransferase P300 is also reported at pluripotency-related genes. The bromodomain of BRD4 works with P300 to increase its catalytic activity, recruiting BRG1 to alter the chromatin structure of pluripotency genes in ESCs [54,55], and OCT4 actively cooperates with BRD4 to define undifferentiated states [56]. Live-cell single-molecule fluorescence microscopy studies reveal that dynamic bromodomain-mediated activation hubs enable PBAF domains to reorganize nucleosomes [57].

Interestingly, a recent study in mouse ESCs identified the bookmarking role of BAF subunits SMARCE1 and SMARCB1 [58]. These subunits bookmark cell-type-specific and mitosis-G1 transition genes, ensuring their prompt reactivation post-mitosis and contributing to cell fate memory. These findings reveal a complex interplay between pioneer factors like OCT4 and BAF subunits in regulating pluripotency maintenance. Beyond pluripotency, similar interactions between pioneer factors and BAF subunits in regulating cell type specificity have been observed in other systems, such as the role of ASCL1 in neural differentiation [59]. Such TF–BAF interactions are also crucial during tumourigenesis where cancer cells exploit lineage-specific TF-mediated developmental programmes to promote cancer development and progression [60]. For instance, Brg1 is essential for sustaining the oncogenic transcriptional programme in leukaemia cells, particularly by targeting the Myc gene. This regulation involves a cluster of lineage-specific enhancers 1.7 Mb downstream of Myc, binding SWI/SNF components and Brd4. Brg1 maintains transcription factor binding at these distal enhancers, facilitating long-range chromatin interactions with the Myc promoter, ensuring Myc transcriptional activation in leukaemia cells [61]. Among the TFs which bind with SWI/SNF subunits in leukaemia cells is pioneer factor PU.1, which primarily binds to its targets independently of the SWI/SNF and subsequently recruits SWI/SNF to enhance chromatin accessibility for other critical AML regulatory factors, such as RUNX1, LMO2 and MEIS1 [62] (figure 3).

These findings, along with other studies, demonstrate that SWI/SNF-dependent distal enhancers are crucial for regulating gene expression associated with developmental processes and tumourigenesis [63].

## 5. Role of SWI/SNF complex in organ development

The SWI/SNF complex participates in several biological processes, including development, cell proliferation and differentiation through gene expression regulation, some of which are described below in detail.

**Figure 4.**
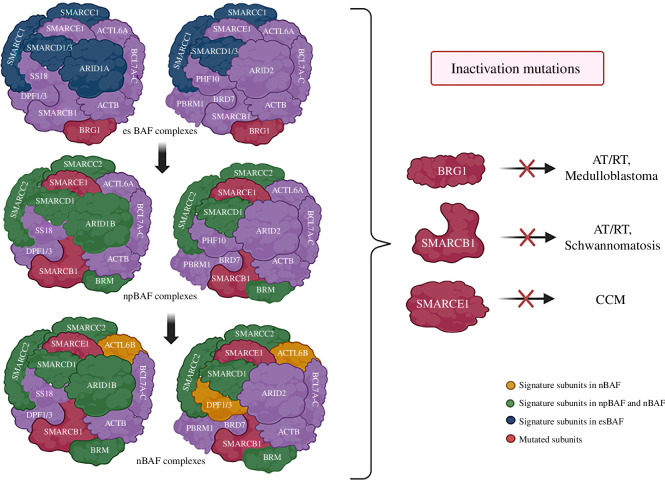
The involvement of SWI/SNF complexes in neurogenesis and brain tumours. The transition of embryonic stem cells BAF (esBAF) to neuronal progenitors BAF (npBAF) occurs with specific subunit switches, namely BRG1, ARID1A and SMARCC1/BAF155 by BRM, ARID1B and SMARCC2/BAF170, respectively. Consequently, the npBAF complexes reassemble into neural BAF (nBAF) complexes. Three significant subunit switches have been linked to the npBAF–nBAF complex transition: DPF1 (BAF45B) or DPF3 (BAF45C) replaces PHF10 (BAF45A), ACTL6A (BAF53A) replaces ACTL6B (BAF53B) and CREST replaces SS18. Additionally, the SMARCC1 homodimer is incorporated in the nBAF complexes rather than the SMARCC1: SMARCC2 heterodimer in the npBAF complexes, while the SMARCD3 (BAF60B) subunit is absent from both the npBAF and nBAF complexes. Inactivating germline mutations in various SWI/SNF subunits, namely SMARCB1, SMARCE1 and BRG1, have been found frequently in various brain tumours. AT/RT, atypical teratoid/rhabdoid tumour; CCM, clear cell meningioma.

Previous investigations have revealed that switches between SWI/SNF subunits are essential in determining neural cell fate. The differentiation of ESCs to neuronal progenitors (NP) co-occurs with the switching of esBAF to npBAF-specific subunits: BRG1, ARID1A and SMARCC1/BAF155 by BRM, ARID1B and SMARCC2/BAF170, respectively (figure 4) [64–66]. Consequently, neural progenitor cells transition to neuronal fate in the cerebrum, leading to the re-composition of the corresponding npBAF complexes into neural BAF (nBAF) complexes. The npBAF–nBAF complex transition has been associated with three major subunit substitutions: PHF10 (BAF45A) is replaced with DPF1 (BAF45B) or DPF3 (BAF45C), ACTL6A (BAF53A) with ACTL6B (BAF53B) and SS18 with CREST [67,68]. Acquiring the ACTL6B (BAF53B) subunit in the nBAF is necessary to regulate cell cycle exit in maturing neurons [69]. Moreover, the nBAF complex also incorporates the SMARCC1 homodimer rather than the SMARCC1: SMARCC2 heterodimer in the npBAF complex while SMARCD3 (BAF60C) subunit was upregulated several folds in neurons relative to ES cells [67,70–72]. Recent studies have revealed that ARID1A plays a crucial role in the proliferation and differentiation of neural stem/progenitor cells (NSPCs) during cortical development in mice. Selective deletion of ARID1A decreases cortical thickness in the maturing brain, impairs radial glial cell proliferation, promotes cell mortality during late neurogenesis, and downregulates specific genes responsible for proliferation and differentiation [73]. To emphasize the critical role of the BAF complex in neurogenesis, it has been reported that BAF170 globally suppresses Pax6 target genes, which regulate intermediate and late progenitors crucial for upper layer neuron generation. During early neurogenesis, BAF170 competes with the BAF155 subunit in the mSWI/SNF complex, modulating euchromatin structure and repressing Pax6 target genes by recruiting the REST–corepressor complex to their promoters. Additionally, research has demonstrated that the BAF complex is involved not only in the central nervous system but also in neuron generation in the olfactory system. BAF155 and BAF170 were identified as crucial regulators of olfactory neural stem cell (oNSC) specification, self-renewal and neuronal maturation in the developing olfactory epithelium (OE). It further reveals a novel mechanism where the interaction between BAF155-containing BAF complexes and the transcription factor Pax6 governs OE neurogenesis [68,74].

The process of haematopoiesis involves a series of consecutive stages that produce various differentiated cell types. The development of undifferentiated haematopoietic progenitor cells into mature lymphoid, myeloid and erythroid lineages necessitates transcriptional regulation and epigenetic oversight of lineage-specific genes [75,76]. Studies showed that ARID1A is necessary for normal myeloid and lymphoid lineage differentiation and the maintenance of typical haematopoietic stem cell numbers and function. Critical genes involved in haematopoietic development were impacted by transcriptional alterations caused by ARID1A loss, including CEBPA, CD34, CSF1, IL6RA and GATA2 [77]. Recent studies have also demonstrated that the deletion of ARID2 autonomously impacts HSC formation, diminishing the capacity to reconstitute the lymphoid lineage during bone marrow transplantation. This effect was mediated through perturbed HSC differentiation via inflammatory pathways. However, the absence of ARID2 does not affect steady-state haematopoiesis except for erythropoiesis [78]. Although previous research showed that SWI/SNF complexes are necessary for inflammatory gene activation in macrophages, the role of specific subunits associated with the different SWI/SNF complexes was not elucidated. Genome-wide profiling of nascent transcription and chromatin accessibility after SWI/SNF subunit deletion is needed to better understand the potential diverse roles of the SWI/SNF complex variants in stimulus-dependent transcription [79].

To regulate haematopoietic differentiation, the acute myeloid leukaemia 1 (AML1 or RUNX1) transcription factor creates multi-protein complexes with BRG1 and SMARCB1 that bind to RUNX1 target gene promoters such as GMCSF, IL3, MCSF-R, MIP and P21 during myeloid differentiation [80]. Furthermore, the PHF10 (BAF45A) subunit has recently been shown to be crucial for maintaining adult haemopoietic stem cells and the development of the myeloid lineage. In mice, PHF10 deletion is embryonic lethal. Conversely, deleting PHF10 acutely in adults reduces the number of long-lived repopulating haemopoietic stem cells and committed myeloid progenitors without changing their proliferation rate [81].

Further, ARID1A organizes OCT4 and β-CATENIN binding in the mesodermal progenitor cells in a manner that particularly governs the efficient differentiation of cardiac mesoderm. Upon ARID1A deletion, OCT4 and β-CATENIN were recruited less often at the promoters of key genes for the mesodermal lineage specification, including MESP1 and EOMES, and corresponding lineage-specific genes were downregulated [82]. During the initial stages of heart development, four SWI/SNF-specific subunits, ARID1A, SMARCD3/BAF60C, PBRM1/BAF180 and ARID2, demonstrate high expression levels, which undergo substantial suppression as development and differentiation progress [83]. Notably, silencing Smarcd3 in mouse embryos through RNA interference or knockout of ARID1A and PBRM1 exhibit heart malformation and increased mortality [11,84,85]. Moreover, ARID1A specifically controls the expression of a cluster of genes to regulate cardiac progenitor cells (CPC) differentiation into mature cardiomyocytes by preferentially binding to promoters of *MEF2C*, *NKX2.5* and *BMP10* and recruiting BRG1 to alter chromatin accessibility [86,87]. The significance of ARID1A-containing complexes in cardiac gene regulation has been established through their direct interaction and potential collaboration with subunits of the nucleosome remodelling and histone deacetylase (NURD) complexes in a repressive manner. This occurs by modifying chromatin sites associated with active or repressed gene expression, shifting them between accessible and closed states [83].

Numerous cardiac abnormalities, including single ventricle, constricted outflow tract and inadequate trabeculation, are shown to occur due to RNA interference of SMARCD3 [84]. Notably, SMARCD3 may aberrantly induce cardiomyocyte development in concert with GATA4 and TBX5 [88] in zebrafish, suggesting that it can direct CPC migration to the nascent heart region [89]. Moreover, impairment in cardiac formation and coronary maturation, as well as ventricular septal defect in mouse embryos, result from the deletion of PBRM1 through downregulation of specific target genes; S100A13, RARβ2 and CRABPII in the heart [85].

To our knowledge, few studies have examined the role of BAF/PBAF subunits in liver development. One study found that deleting SNF5 in the mouse liver led to severe hypoglycemia, neonatal mortality, poor energy metabolism and an inability to store glycogen, ultimately hindering the development of hepatic epithelium. Furthermore, transcriptome analysis revealed that 70% of genes normally upregulated during liver development were transcriptionally silenced following SNF5 deletion. These genes include those associated with cell–cell adhesion, gluconeogenesis and glycogen production [90].

## 6. Role of SWI/SNF subunits in organ regeneration

Considered master regulators, SWI/SNF subunits play a crucial role in tissue regeneration, which is the process through which organisms reconstruct lost or injured tissue. A recent investigation focused on pancreatic β-cells highlights the essential role of the ARID1A-containing complex in guiding cell regeneration. Given the abundance of ARID1A in quiescent β-cells, its depletion during pregnancy or post-pancreatectomy suggests that the absence of ARID1A enhances β-cell regeneration by activating the EGF signalling pathway [91].

Embryonic hepatoblasts are the primary progenitor cells in the developing liver, giving rise to hepatocytes, cholangiocytes and intrahepatic bile ducts (IHBD) [92]. The mature liver has an exceptional ability for regeneration; however, less than 2% of hepatocytes and biliary cells develop and grow when the body is in homeostasis. Nevertheless, a substantial proliferative behaviour is elicited upon damage, which helps to replenish both hepatocytes and cholangiocytes [93,94].

Investigations into the role of epigenetic markers in liver regeneration indicate that the quiescent liver could have an epigenetic code that determines its regenerative capability [95]. The shift in the expression of dozens of genes occurs together with the shifting of hepatocytes from quiescent to proliferative. For the liver to continue functioning as it expands, this dynamic transcriptional profile ensures cellular and tissue repopulation while maintaining ancestral identity [96]. Research indicates that deleting the SWI/SNF subunit ARID1A significantly promotes organ regeneration, as demonstrated in a mouse model. After induced damage, mice with the Arid1a gene deleted have significantly improved liver and ear regeneration. In the liver, Arid1a impairment promotes cell proliferation following surgical resection and chemical injury, reduces tissue damage and fibrosis and supports overall organ function [97]. Interestingly, however, another study had shown a positive role of Arid1a on liver regeneration by creating a permissive chromatin state in hepatocytes, enabling them to respond to regenerative signals and express liver-progenitor-like genes [98]. One argument provided for these contradictory findings was that Arid1a has multiple roles in liver regeneration: it facilitates the formation of liver-progenitor-like cells during injury and temporarily hinders proliferation during recovery [98].

BRM and BRG1 ATPase subunits also exhibit differential expression during the liver regeneration process. Such complexes coordinate the alteration of nucleosomes to recruit diverse transcription factors that facilitate gene activation depending on the phase of the cell cycle, the developmental stage and the specific tissue [99]. During mouse liver injury, the SWI/SNF complex features BRG1 as the ATPase subunit, with complexes involving BRM potentially becoming predominant towards the end of the injury stage and the start of regeneration. Later, BRG1-containing complexes regain prominence in the regeneration phase. As a result, BRG1 regulates genes involved in cellular proliferation, while BRM controls genes associated with cellular differentiation and growth restriction [99,100].

## 7. Role of SWI/SNF remodellers in carcinogenesis

The prevalence of inactivating mutations, including nonsense, frameshift and deletion mutations, in various SWI/SNF subunits has recently been observed in numerous malignancies. This suggests that these complexes play a widespread role in tumour suppression across different types of cancers [101]. At least nine distinct SWI/SNF subunits have been reported to be frequently mutated in different tumours (table 1). These mutations are reported in about 25% of all malignancies despite the fact that the exact molecular mechanisms behind their role in tumourigenesis are still unclear [14].

**Table 1.** Frequently mutated SWI/SNF subunits in different tumours. SWI/SNF subunits have been reported to be frequently mutated in different tumours. These mutations are observed in about 25% of all malignancies.

SWI/SNF subunit	cancer	reference
SMARCB1	atypical teratoid/rhabdoid tumour (AT/RT)	[102]
	schwannomatosis	[103]
BRG1	atypical teratoid/rhabdoid tumour (AT/RT)	[104]
	medulloblastoma	[105]
	acute myeloid leukaemia (AML)	[61]
	lung cancer	[106]
SMARCE1	clear cell meningioma (CCM)	[107]
BCL7A	diffuse large B-cell lymphoma (DLBCL)	[108]
ARID1A	acute myeloid leukaemia (AML)	[109]
	varian clear cell carcinoma	[110]
	varian endometrioid carcinoma	[111]
	hepatocellular carcinoma (HCC)	[112]
	gastric adenocarcinoma	[113]
	cholangiocarcinoma	[114]
ARID1B	acute myeloid leukaemia (AML)	[115]
ARID2	acute myeloid leukaemia (AML)	[116]
	hepatocellular carcinoma (HCC)	[117]
	non-small cell lung cancer	[118]
	gastric adenocarcinoma	[113]
	melanoma	[119]
PBRM1	clear cell renal cell carcinoma	[120]

The discovery of biallelic, truncating mutations in the SMARCB1 (INI1, hSNF5, BAF47) gene in atypical teratoid/rhabdoid tumour (AT/RT), a particularly aggressive paediatric cancer, gave the first evidence of their role in carcinogenesis (figure 4) [102]. A small minority, approximately 2% of AT/RT cases have intact SMARCB1, instead contain inactivating mutations in BRG1 on chromosome 19p13.2 [121]. Individuals with BRG1-defective RTs typically manifest in early childhood, with a median age of 9 months (range 0−28 months), often exhibiting germline mutations [122]. Nearly all BRG1 genetic alterations lead to the complete loss of its expression, primarily due to nonsense mutations, deletions or copy-neutral loss of heterozygosity [104].

Moreover, SMARCB1 mutations in the germline may predispose individuals to both malignant rhabdoid tumours and schwannomatosis, a rare genetic disorder that results in multiple tumours called schwannomas, but they seldom occur simultaneously in the same family [103]. Almost all SMARCB1 mutations linked with AT/RT are truncating mutations or deletions that result in total knockout of the SMARCB1. By contrast, SMARCB1 mutations reported in schwannomatosis are primarily non-truncating, missense or splice-site mutations and in-frame deletions, thus probably leading to mutant protein with residual function [1,123].

A rare cancer called clear cell meningioma (CCM) is marked by the biallelic inactivation of the SMARCE1/BAF57 gene [107]. Individuals with germline SMARCE1 loss-of-function mutations in one allele, with a second hit that inactivates the other SMARCE1 allele, renders them at high risk of developing paediatric CCMs [124]. Germline mutations might be deletion, insertion or inversion, although heterozygous frameshift or nonsense point mutations accounted for the majority [125].

Moreover, medulloblastoma is associated with recurrent mutations in members of the SWI/SNF family, including BRG1, primarily limited to WNT and G3 subgroups [105]. Epigenetic antagonism between Polycomb Repressor complexes (PRC2) and SWI/SNF contributed to evaluating PRC2 inhibitors in paediatric malignancies with SWI/SNF mutations. As the catalytic component of the PRC2, EZH2 catalyses the trimethylation of histone 3 lysine 27 (H3K27) at target gene promoters, resulting in gene silencing [126].

In addition, alterations in the subunits of the SWI/SNF complex, particularly in ARID1A/ARID1B/ARID2, BRM/BRG1 and BCL7A (B-cell CLL/lymphoma 7 protein family member A), are prevalent in the onset or progression in a broad range of myeloid and lymphoid blood malignancies [127]. Most genetic changes result in loss-of-function-mutations, reflecting tumour suppressor function [128]. Deletion of 27 amino acids occurs in the N-terminal domain of BCL7A in diffuse large B-cell lymphomas (DLBCL) due to a recurrent mutational hotspot at the splice donor site of intron 1, which prevents the SWI/SNF complex assembly [108].

In two human acute myeloid leukaemia (AML) cell lines, HL-60 (APL) and THP-1 (*KMT2A*-rearranged), a recent investigation suggested that ARID1A may function as a barrier to unregulated cell division. Reduced ARID1A expression specifically inhibited apoptosis and increased AML cells’ potential to proliferate via the TGF-β1/SMAD3 pathway [109]. Similarly, a recent study found that in a mouse model of *KMT2A*-rearranged AML, deletion of ARID1B facilitated both AML onset and progression [116]. Surprisingly, the researchers could not uncover any phenotypic changes after knocking down ARID1B *in vitro* [115]. Unlike ARID1B, the effects of ARID2 could vary depending on the stage of AML. Specifically, ARID2 deletion increases leukaemogenesis in the early phases of AML, while ARID2 activity is subsequently necessary for AML maintenance. Comparable with ARID1B, ARID2 knockout results in phenotypic changes that can only be observed *in vivo* [116].

By contrast to the tumour suppressor role of the BRG1 subunit in different types of malignancies, its unusual role as a tumour-supporting gene in some cancers is becoming apparent. BRG1/SMARCA4-containing SWI/SNF complexes may be necessary for AML maintenance and may provide novel avenues for intervention [61,129]. Indeed, SMARCA4-containing SWI/SNF complexes have been shown to specifically regulate MYC oncogene in AML [61]. Comprehensive evidence of this regulation has been provided by the human cell line ME-1 (AML bearing the *CBFB-MYH11* fusion gene) [130]. On the other hand, the involvement of BRM in haematological malignancies remains unclear. A recent study found that the viability of a variety of AML cell lines was substantially decreased by the simultaneous loss of BRM and BRG1 function, which was achieved either by knock-down approaches or the use of allosteric dual inhibitors like BRM011 and BRM014 [131].

Recent studies on lung cancer have shown that over 20% of lung cancer patients exhibit recurring changes in several SWI/SNF genes, some of which have a substantial correlation with a poor prognosis, indicating an essential function of SWI/SNF [132]. Another recent work demonstrates that the deletion of BRG1 disrupts the SWI/SNF complex, decreasing chromatin accessibility at lung lineage-specific DNA motifs and eventually promoting tumourigenesis. It suggests that throughout the progression of lung cancer, the SWI/SNF complex, via BRG1, functions as a checkpoint for lineage-specific transformation and metastasis [106].

Hepatocellular carcinoma (HCC) is the primary liver cancer that accounts for more than 90% of all major liver cancers. Hepatocellular carcinoma develops in around 85% of cirrhotic livers. HCC is currently the fourth most frequent form of cancer globally and the second leading cause of cancer mortality in males after lung cancer [133]. However, a significant factor driving the increase in liver cancer in developed nations will be the prevalence of non-alcoholic fatty liver disease (NAFLD), combined with metabolic syndrome and obesity, both of which increase the risk of liver cancer [134].

The involvement of epigenetic modulators in tumour propagation has been established since the advent of cancer genome-wide sequencing, that had identified large genetic variants crucial for regulating chromatin structure, including ARID1A and BRCA1-associated protein 1 (BAP1) as well as TERT, TP53 and CTNNB1 are found to be the most frequently mutated genes in HCC [112]. ARID1A, which is noted to be a tumour suppressor protein, is frequently mutated or deleted in HCC and associated with poor prognosis in patients with increased invasion and metastasis. It is also closely related to tumour immune cell infiltration [135]. The loss of ARID1A can stimulate tumourigenesis due to its requirement for the differentiation-associated cell cycle arrest [136], and activating the PI3K–AKT signalling pathway, which alters the expression of several transcription factors, including the MYC gene [137,138]. Similarly, recent findings also support the concept that the lack of ARID1A might promote the progression of HCC by upregulating MYC [139].

Many findings pointed to a more nuanced involvement for ARID1A in HCC, and distinctive SWI/SNF components can be oncogenic in different scenarios relying on context-specific parameters such as timing, tissue location, existence of collaborating mutations and dosage [140]. Nevertheless, the fact that ARID1A was significantly elevated in primary tumours yet not in metastatic tumours among certain HCC patients suggests that ARID1A might be depleted upon onset [141,142]. Although ARID1A upregulation exacerbates tumour development, mice having liver-specific homozygous or heterozygous ARID1A deletion were imperviable to tumour initiation [143]. However, homozygous or heterozygous ARID1A deletion in preexisting tumours enhances invasion and metastasis [144]. Recent studies also propose an epigenetic function for ARID1A in regulating lipid homeostasis in the liver. It exhibits direct control of target genes in the lipogenic and fatty acid oxidation pathways by interacting with promoter regions and affecting the accessibility of transcriptional machinery. ARID1A reduction leads to nonalcoholic steatohepatitis (NASH) [145,146]. As the other ARID component of the SWI/SNF complex, ARID2 binds DNA regardless of the sequence. On the basis of its position in the PBAF complex, ARID2 has been implicated in the control of tissue-specific gene expression [147]. There are reports of ARID2 loss-of-function mutations in a wide variety of human malignancies, such as melanoma, gastric adenocarcinoma, non-small cell lung cancer and hepatocellular carcinoma, suggesting its tumour suppressor function [113,117–119,148].

In recent investigations, ARID2 expression was reported to be considerably decreased in metastatic HCC tissues, and it was linked with poor prognosis in HCC patients and significantly correlated with tumour metastasis [149]. According to data, the interruption of the DNA repair machinery, nucleotide excision repair (NER), due to ARID2 deletion could induce DNA damage, increasing the risk of developing cancer and hypermutations [150]. Additionally, ARID2 reduction hastens the G1/S transition, which was also coupled with increased levels of cyclin D1, cyclin E1, CDK4 and retinoblastoma protein phosphorylation (Rb), suggesting that ARID2 suppresses hepatoma cell-cycle advancement and tumourigenesis by targeting the Rb-E2F signalling pathway [151].

Cancer cells acquire metastatic features via the epithelial–mesenchymal transition (EMT), mediated by a confined set of transcription factors, primarily members of the SNAIL, TWIST and ZEB families [152]. Previous findings have highlighted that ARID2 restricts the metastatic spread of HCC cells and hinders the epithelial–mesenchymal transition (EMT) by recruiting DNMT1 to the SNAIL promoter, leading to DNA methylation at the SNAIL promoter CpG island. Thus, ARID2 mutations interrupting its C2H2 domain could not recruit DNMT1 to the SNAIL promoter, resulting in reduced methylation of its promoter, linked to vascular metastasis and poor prognosis in HCC patients [153].

A recent study of NAFLD discovered that ARID2 stimulates JAK2 ubiquitination, which is achieved via recruiting CARM1 to enhance H3R17me2a levels at the NEDD4L promoter and upregulate NEDD4L expression, a novel E3 ligase for JAK2. This increased JAK2 ubiquitination suppresses the JAK2–STAT5–PPARγ signalling pathway and reduces hepatic steatosis [154]. The implication of ARID2 mutations and, more broadly, the disruption of chromatin regulatory mechanisms in cancer suggests the necessity of anticancer therapies targeting chromatin-associated proteins.

ARID1A depletion in cholangiocarcinoma results in cellular proliferation, cell cycle escape, senescence and extensive alterations in heterochromatin. A new finding demonstrated that the failure to activate the TGF–SMAD4 tumour suppressor pathway is the underlying cause of the biliary proliferative response induced by KRAS mutation and ARID1A depletion [114]. Similarly, a next-generation sequencing analysis of cholangiocarcinoma found chromatin modification among the most affected processes, along with ARID1A and PBRM1 being the most frequently mutated genes [155].

In conclusion, the data gathered have established the SWI/SNF chromatin remodeller as a major human tumour suppressor complex and have opened the door for the researchers to elucidate the processes by which the loss of SWI/SNF subunits promote carcinogenesis.

## 8. Convergence of the molecular mechanisms during development and carcinogenesis by SWI/SNF

**Figure 5.**
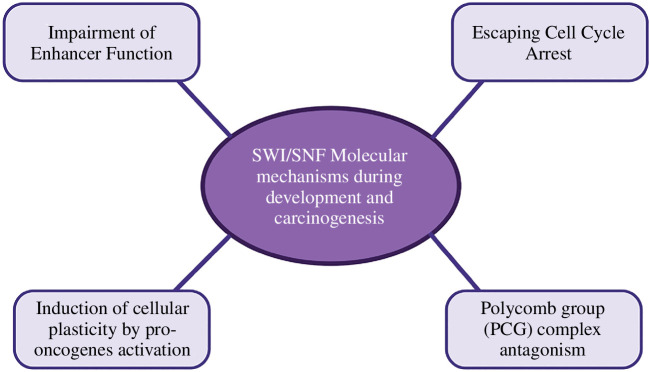
Convergence of the molecular mechanisms during development and carcinogenesis by SWI/SNF. The onset and development of carcinogenesis are associated with particular molecular signatures linked to SWI/SNF mutations, such as the disruption of SWI/SNF activity at enhancers, evasion of G0 cell cycle arrest, induction of cellular plasticity through pro-oncogene activation, and antagonistic interactions with the Polycomb group (PcG) complex.

The mechanisms of tumour suppression by the SWI/SNF complex and the degree to which mutations in SWI/SNF subunits contribute to carcinogenesis are ongoing research areas. Nevertheless, a consensus is evident in various cancers as numerous studies have explored the impact of malfunctioning subunits within the SWI/SNF complex, revealing common patterns in its functionality (figure 5). Interrupted SWI/SNF activity at enhancers involved in lineage-specific development and specification is observed often [63,156,157]. By contrast, another pattern has emerged demonstrating that SWI/SNF mutations promote escaping G0 cell cycle arrest [158–160]. These are probably not the sole mechanisms through which SWI/SNF mutations manifest their effects. Recent findings have highlighted [48,161] Polycomb group (PcG) complex antagonism with SWI/SNF subunits, apart from their impact on well-established cancer-promoting pathways and oncogenic transcription factors, as further detailed below.

### 8.1. Impairment of enhancer function

SWI/SNF complex subunits have been demonstrated to work with signal-responsive transcription factors to activate enhancer function in a cell type-specific manner [48]. They promote enhancer-associated gene expression programmes required for cell differentiation and lineage commitment [156]. Studies show that Brg1 coordinates B cell development by acting at different developmental stages. In particular, BRG1 provides access to a broad enhancer repertoire for transcriptional regulators directly connected with a B lineage-specific transcriptional profile at the earliest stages of B cell development. BRG1 was required to integrate distal and proximal variable regions and accessibility to IGH locus transcription factor-binding sites in committed pro-B cells. By allowing EBF1, IKAROS and PAX5 access to a distally located MYC super-enhancer, BRG1 regulated pro-B cell proliferation and halted premature pre-B cell differentiation [157]. Moreover, BRG1 and the transcription factor OLIG2 selectively bind to the enhancers of genes that regulate oligodendrocyte development. OLIG2 recruits BRG1, containing a bromodomain, to recognize active histone acetylation marks such as H3K27Ac and induce the generation of key differentiation activators, notably MRF and SOX10 [162].

Genetic changes inevitably result in dysregulated transcriptional patterns in cancer, which are frequently mediated via the remodelling of the enhancer landscape [163]. Notably, it has been suggested that super-enhancers, vast clusters of enhancers with high cumulative H3K27Ac contents, regulate genes essential for establishing cell identity and those involved in the malignant state of cancer [164]. In prostate cancer, for instance, degradation of SWI/SNF ATPase impairs the super-enhancer and promoter looping interaction and binding of a set of transcription factors that promote cellular growth to cis-regulatory elements [165].

Similarly, an animal model of colon cancer has revealed that ARID1A often directs SWI/SNF complexes to enhancers, where they collaborate with transcription factors (TFs) to induce gene expression. In ARID1A-deficient cells, ARID1B maintains SWI/SNF function, but deficiencies in SWI/SNF targeting and control of enhancer activity result in significant dysregulation of gene expression [166]. Furthermore, ARID1A works as a cofactor at enhancers taken up by AP1 transcription factors operating downstream of the MEK/ERK pathway in colorectal cancer (CRC). Loss of ARID1A consistently resulted in a disruption of KRAS/AP1-dependent enhancer function and a decrease in expression of the corresponding target genes such as EREG, F3 and JAG1 [167].

Additionally, BRM containing SWI/SNF complex, which includes the actin-related BAP55 subunit, is necessary for nucleosome turnover and the enhancer accessibility required for the Notch response. Notch signalling modulates nucleosome turnover at target enhancers and promotes the inclusion of the histone variant H3.3 [35,168]. Moreover, the SMARCB1 C-terminal domain has a basic helix that directly binds to the nucleosome acidic patch. Mutations that hinder this binding interrupt SWI/SNF-mediated nucleosome remodelling activity and enhance DNA accessibility [169].

Rhabdoid tumours, which can develop in the brain, kidney and soft tissues, are solely driven by the deletion of SMARCB1 (SNF5) subunit [170]. SMARCB1 stabilizes the SWI/ SNF complex, allowing it to bind and maintain enhancer generation and functioning. The deletion of SMARCB1 causes a significant decrease in the number of SWI/SNF complexes, reaching insufficient levels to sustain proper enhancer activity. The remaining SWI/SNF complex then preferentially attach to super-enhancers. While typical enhancers are required for differentiation, super-enhancers have been related to maintaining existing cell identity. Thus, these findings point to a scenario in which the absence of SMARCB1 hampers the function of enhancers, which are essential for differentiation, but leave super-enhancers, which are responsible for maintaining existing cell identity, substantially untouched. As a result, in some proliferative progenitor cell types, diminished enhancer activity with super-enhancer retention may induce tumourigenesis by maintaining cells in a poorly differentiated and highly proliferating state [171].

### 8.2. Escaping cell cycle arrest

To varying degrees, SWI/SNF tends to control cell cycle progression, development and differentiation, senescence, preservation of cell identity and virtually all major cellular activities [74,172]. For instance, the involvement of SWI/SNF in myoblast development is critical. Myoblasts rely on BRG1 to guide them through the cell cycle. Myoblast survival and expansion are facilitated by transcription factor Pax7, while BRG1 is essential to sustain steady Pax7 expression. In addition, ARID1A plays a crucial role in cell proliferation by binding to and rearranging the chromatin around the Pax7 promoter [173,174]. It is interesting to note that BRG1 and BRM play distinct roles at different stages of muscle differentiation. While BRG1 is necessary for activating muscle gene transcription in the early stages, BRM is essential for causing cell cycle arrest before muscle genes are activated [173,175]. Remarkably, BRM’s role in myogenic differentiation depends on its capacity to directly block transcription of cyclin D1, an established activator of the G1–S phase transition during myoblast proliferation and a distinct inhibitor of muscle development [175,176].

Moreover, loss of BRM in mouse keratinocytes has been linked to UV-induced skin and corneal carcinogenesis since it facilitates rapid entry into the G1 phase. Following the entry into G1-phase cell cycle arrest, cyclin D1 is degraded by proteolytic cleavage, and this process is then sustained by a p53-mediated transcription regulation, which increases the expression of key regulatory genes, such as p21 [177,178]. Similarly, the effects of BRM and BRG1 on the cell cycle checkpoints in murine lung cancer have been demonstrated. BRG1 is crucial for activating the tumour suppressor protein retinoblastoma (RB1). Loss of BRG1 causes a shift in RB1 phosphorylation via the downregulation of cyclin-dependent kinases (CDKs) and G1 cyclins through the GSK3β pathway, which governs the localization of p21. On the other hand, BRM complexes have been linked to TP53, although the clinical relevance of this connection is not yet clear [179].

The levels of SMARCD3, have shown a correlation with breast cancer risks. The expression of SMARCD3 was associated with hormone-positive (ER+) breast cancer and correlated with differential long-term disease-free survival. SMARCD3 depletion results in lower proliferation rates, increased endoreplication and unresolved DNA damage, suggesting its potential role as a tumour suppressor and a specific prognostic biomarker for breast cancer [180,181]. Interestingly, silencing Smarcd3 in EpCAM-breast cancer cells promotes a robust transition from mesenchymal to epithelial phenotype, while its expression in mammary epithelial cells induces EMT. Smarcd3 drives mesenchymal-like changes through Wnt5a upregulation, with EMT reversible by Wnt5a inhibition, highlighting its role in epigenetically regulating EMT via WNT signalling. These contradictory effects of Smarcd3 in breast cancer suggest a more complex role of this subunit at specific stages of carcinogenesis [182].

By contrast, the BRG1 subunit is found at the promoters of genes, including CDK4, LIG1 and NEIL3, whose transcription is regulated by cell cycle progression and is heavily acetylated by EP300 in dividing breast cancer cells. Cell cycle arrest in G1 caused by CDK4/6 inhibition mimics the effect of long-term BRG1 inhibition on chromatin structure. Such findings imply that BRG1 may regulate gene transcription by boosting the expression of genes involved in cell cycle progression in the breast cancer cells examined [183].

The recently identified functions of ARID1A in the DNA damage response (DDR) have been elucidated through the actions of two signalling molecules that recognize such incidents; ATM (ataxia-telangiectasia mutated) and ATR (ATM and Rad3-related). A single-strand DNA break (SSB), DNA replication stress and DNA-end resection activate ATR, while a double-strand DNA break (DSB) often activates ATM, which generates a single-strand DNA region during DSB repair [184,185]. ATR was found to interact with ARID1A to recruit it to DNA DSB sites, resulting in the recruitment of the ATPase subunit of the SWI/SNF complex to DNA damage sites. The G2/M DNA damage checkpoint is hampered by ARID1A loss [186]. In particular, suppression of ARID1A impairs NHEJ by reducing the recruitment of NHEJ proteins to the DSB sites, such as KU70/KU80 and the ATPase subunit of SWI/SNF. Hence, ARID1A-containing SWI/SNF complexes allow several DNA repair machineries to access DNA damage sites efficiently [187].

These data, taken together, demonstrate that chromatin modification by the SWI/ SNF complex serves a role in regulating cell cycle and differentiation and DNA repair. While disruption of its subunits results in exiting cell cycle arrest and metastasis.

### 8.3. Induction of cellular plasticity through pro-oncogene activation

More notably, recent data have revealed that specific SWI/SNF mutations substantially drive malignancies through a profound oncogenic mechanism. This pattern adds a crucial dimension to our understanding of the origins of these tumours. Preliminary evidence suggests that interactions between SWI/SNF and oncoprotein transcription factors such as MYC, AP-1, TAZ, YAP or mTOR are implicated in various cancers. The MYC protein family is a set of basic-helix-loop-helix-leucine zipper transcription factors that play a significant role in carcinogenesis [188]. It has been established that MYC interactions with several chromatin remodellers, including SWI/SNF complex, contribute to its efficiency in regulating gene expression related to metabolism, angiogenesis, invasion, tumour microenvironment, protein synthesis and cell proliferation. Research has shown that SMARCB1 inhibits MYC activity by limiting its ability to access chromatin, diminishing target gene transcription [189,190].

An improved perspective of the MYC–SNF5 interaction indicates that SNF5 deletion in tumours, particularly in malignant rhabdoid tumour (MRT), leads to uncontrolled MYC activity with the help of SNF5 depleted SWI/SNF complex and describes how MYC target genes are upregulated in SNF5-deleted cancers. Moreover, the findings suggest that MYC is a primary driver of oncogenic pathways in MRT and that MYC inhibitors, when accessible, might be applied to treat SNF5-deleted cancers [191]. Thus, the absence of SNF5 does not result in the SWI/SNF complex becoming inactive rather, it facilitates oncogenesis due to the activity of the residual SWI/SNF complex that contains BRG1 [192].

Similarly, another study suggests that MYC directly interacts with other SWI/SNF subunits, such as SMARCC1. It shows that MYC and SNF5 compete for binding to SMARCC1 and SNF5 efficiently inhibits MYC from recognizing its binding site on the SMARCC1 subunit. The finding that MYC can precisely interact with SMARCC1 is relevant due to the fact that SMARCC1 homodimers act as an initial SWI/SNF intermediate by which further SWI/SNF subunits, along with SNF5 can integrate, enabling the complex to react with non-SWI/SNF proteins like MYC [161,190]. Typically, SNF5 would target normal SWI/SNF complexes and possibly AP-1 to enhancers related to differentiation and development. In malignant rhabdoid tumour (MRT) cells, the SWI/SNF selection of enhancers is compromised when SNF5 is lost [161].

Moreover, one of the most prevalent genetic events during the onset and progression of hepatocellular carcinoma (HCC) is c-MYC overexpression [193]. Recent research offers the first *in vivo* proof of BRG1 oncogenic involvement throughout the development of hepatocarcinogenesis that is context and gene dependent [194].

Many different genes are regulated by the transcription factor activator protein 1 (AP-1). It forms several homo or heterodimers with members of the *FOS*, *JUN*, *MAF* and *ATF* multigene families of proteins that possess a highly conserved basic leucine zipper domain (bZip), serving for dimerization and as the basic region in interaction with specific DNA motifs [195]. Studies suggested that direct nucleosome binding by AP-1 as a pioneer factor ultimately directs the BAF complex to these confined regions. The BAF complex then modifies chromatin and stabilizes the pJUN-nucleosome interaction. Other studies have shown that the BAF complex attracts histone acetyltransferases, which results in the enrichment in H3K27Ac on nucleosomes [196]. While AP-1 proteins can function independently as tumour suppressors or oncogenes, they primarily serve as key drivers of upstream oncogenic processes. For instance, dysregulated signalling in the MAPK pathway can increase the expression of several AP-1 constituent genes [197].

YAP is a key regulator of hepatocyte proliferation, regulating the size and regenerative capability of the liver. The ARID1A-containing SWI/SNF complex has been demonstrated to inhibit the pro-oncogenic transcriptional coactivators TAZ and YAP. Researchers established that YAP/TAZ are crucial for unveiling the impacts of SWI/SNF inactivation, including cell proliferation, acquiring stem cell-like features and liver carcinogenesis. It suggests that for enabling YAP/TAZ responses, two criteria must be met: enhancement of YAP/TAZ nuclear accumulation by the loss of Hippo signalling and reduction of ARID1A of SWI/SNF complex through genetic mutations to induce cell plasticity and tumourigenesis [198]. Moreover, it was reported that mTOR complex 1 (mTORC1) promotes the ubiquitination and proteasomal degradation of the ARID1A subunit. Thus, the mTORC1–ARID1A axis induces oncogenicity due to YAP-dependent transcription, which promotes liver oncogenic proliferation, indicating that the YAP pathway is a significant oncogenic driver of liver cancer [199].

### 8.4. Polycomb group complex antagonism

Polycomb-group (PcG) are evolutionarily conserved proteins involved in the irreversible and inherited silencing of gene expression. PcG repressive complex 2 (PRC2) is a multimeric complex that deposits or binds to specific histone modifications, such as H3K27me3 and H2AK119ub1, to hinder gene activation and retain repressed chromatin domains [200]. EZH2, the catalytic subunit of PRC2, is upregulated in various cancers and is frequently associated with accelerated tumour growth and a poor prognosis [201].

As per the previous reports, a disrupted epigenetic antagonistic relationship between the SWI/SNF complex and PRC2 primarily drives the early onset of tumour development following SNF5 loss. In SNF5-deficient malignancies, PcG targets are extensively H3K27-trimethylated and repressed, where the deletion of the SNF5 tumour suppressor leads to increased expression of EZH2 [126]. Recent studies have further added to this observation, showing that rapid BAF depletion shifts the Polycomb repressive complexes, PRC1 and PRC2 from densely occupied regions, such as Hox clusters, to sparsely occupied regions typically suppressed by BAF causing their decompaction, gain of active epigenomic features and transcriptional de-repression [202].

Thus, the narrative surrounding SWI/SNF and cancer takes an intriguing trajectory as it intersects with EZH2 and oncogenic transcription factors. This connection holds significant implications for the potential investigation and, hopefully, future management of these tumours.

## 9. Targeting SWI/ SNF mutations: a promising cancer therapy

For many years, oncologists have predominantly relied on non-specific or inadequately targeted treatments like chemotherapy, which often causes significant harm to healthy tissues. The recent discovery of new genetic or epigenetic vulnerabilities specific to cancer, including components of the SWI/SNF complex, has the potential to improve precision medicine and the development of targeted anti-cancer therapies [203].

Despite some glioblastoma (GBM) patients initially responding to the DNA alkylating drug temozolomide (TMZ), a significant majority eventually develop therapeutic resistance, leading to the recurrence of brain tumours. BRG1 is highly expressed in both GBM tumour tissue and GBM cells cultured *in vitro* and is crucial for maintaining the stem cell-like properties of GBM cancer stem cells (GSCs) [204]. PFI-3, a small molecule inhibitor of the BRG1 bromodomain, increases the susceptibility of GBM cells to temozolomide [205]. Moreover, further development of structurally related analogues (SRAPs) of PFI-3, exhibited greater efficacy than PFI-3 in inducing antiproliferation and cell death in GBM cells [206]. These results demonstrated that the BRG1 subunit of the SWI/SNF plays a critical role in regulating sensitivity to TMZ.

ARID1A is mutated in over 50% of ovarian clear cell carcinomas. Using a screen of small-molecule epigenetic inhibitors, Bitler *et al*. identified an inhibitor of EZH2. Their study demonstrates that inhibiting the EZH2 methyltransferase acts in a synthetic lethal manner in ARID1A-mutated ovarian cancer cells, with ARID1A mutations correlating with the response to the EZH2 inhibitor [207]. These findings suggest that pharmacological inhibition of EZH2 could be a novel treatment strategy for cancers with ARID1A mutations. However, resistance to EZH2 inhibitors are observed in ARID1A mutant cancers and recent data suggest that the switch of the SWI/SNF catalytic subunits from SMARCA4 to SMARCA2 underlies the acquired resistance to EZH2 inhibitors [208]. SMARCA4 loss leads to the upregulation of anti-apoptotic genes in EZH2 inhibitor-resistant cells. Consequently, EZH2 inhibitor-resistant ARID1A-mutated cells become highly sensitive to BCL2 inhibitors like ABT263 suggesting that BCL2 inhibition alone or in combination with EZH2 inhibition represents an improved therapeutic strategy for ARID1A-mutated cancers.

Importantly, Tazemetostat has become the first epigenetic therapy approved by the FDA for solid tumours that targets EZH2 in sarcomas with inactivating mutations in SMARCB1 [209].

In the case of HCC, loss of ARID1A prevents glucose deprivation-induced cell death. One of the targets of ARID1A is ubiquitin specific peptidase 9 X-linked (USP9X) gene where AIRD1A recruits histone deacetylase to the promoter of USP9X, resulting in downregulation of USP9X and its target, protein kinase AMP-activated catalytic subunit α2 (PRKAA2). Loss of ARID1A led to increased levels of H3K9 and H3K27 acetylation at the USP9X promoter, upregulating the expression of USP9X and PRKAA2, which mediated the adaptation of tumour cells to glucose starvation [210]. Thus, targeting of USP9X-adenosine 5'-monophosphate-activated protein kinase (AMPK) axis could be a novel therapeutic strategy in HCC patients with the ARID1A mutation.

Addressing the increasing incidence of cancers will demand innovative surveillance and preventive strategies. Moreover, the development of more effective systemic treatments is essential. This includes targeting specific vulnerabilities of SWI/SNF mutant cancers, such as the synthetic lethality observed with EZH2 inhibitors. Combining therapies that inhibit BCL2 or other survival pathways with these targeted treatments could significantly improve outcomes. Research into the mechanisms of resistance and potential combination therapies will be crucial in developing comprehensive treatment strategies for these cancers.

## 10. Conclusion and future perspective

The role of epigenetic modulators in tumour progression has been well-established since the advent of cancer genome-wide sequencing, particularly highlighting the extensive tumour-suppressive functions of SWI/SNF subunits. Our understanding of the molecular mechanisms, the importance of various SWI/SNF subunits in development and organ regeneration, and their involvement in the progression of multiple malignancies has significantly advanced in recent years. Disruptions in SWI/SNF activity at enhancers, evasion of G0 cell cycle arrest, induction of cellular plasticity through pro-oncogene activation, and antagonism of Polycomb group (PcG) complexes are key molecular mechanisms that can be targeted therapeutically in cancers caused by SWI/SNF inactivation. Given the wide range of tumours associated with SWI/SNF mutations, further research to target residual SWI/SNF complexes also holds significant potential for advancing cancer therapy.

The dynamic subunit combinations of SWI/SNF add another level of complexity, which bears important implications for mechanistic, phenotypical and clinical investigations. While it has become apparent that inactivating mutations affecting particular SWI/SNF subunits might provide specific dependencies on distinct genes or pathways, it is still unclear if any general correlations apply to all SWI/SNF associated malignancies, an important subject and an ongoing field of research. Although SWI/SNF complexes have genuine tumour-suppressor functions, there is concern that targeting specific subunits could hasten the development of cancer rather than suppress it. This is because residual complexes might gain new, potentially oncogenic functions [211]. High-throughput screening assays are effective in identifying synthetic lethality of dependencies or similar relations. However, it is yet unknown if dependencies found using cell lines would be sufficiently potent to be applied as therapeutic targets in clinical trials.

Inevitably, there will be further advancements in the coming years, which are expected to provide new insights into novel therapeutic approaches for SWI/SNF mutant malignancies.

## Data Availability

This article has no additional data.
